# Supplementary material for: Aspergillus brasiliensis E_15.1: A Novel Thermophilic Endophyte from a Volcanic Crater Unveiled through Comprehensive Genome-Wide, Phenotypic Analysis, and Plant Growth-Promoting Trails
Source: J Fungi (Basel). 2024 Jul 25;10(8):517. doi: 10.3390/jof10080517 (PMC11355416; doi:10.3390/jof10080517)
Supplement: Supplementary file 1 [file jof-10-00517-s001.zip › jof-3073222-supplementary.pdf]

Aspergillus brasiliensis E_15.1: A Novel Thermophilic Endophyte from a Volcanic Crater Unveiled through Comprehensive Genome-Wide, Phenotypic Analysis, and Plant Growth-Promoting Trails

Amanda Martirena-Ramírez [1], José Germán Serrano-Gamboa [1], Yordanis Pérez-Llano [2], Claribel Orquídea Zenteno-Alegría [3,4], Mario León Iza-Arteaga [1], María del Rayo Sánchez-Carbente [1], Ana María Fernández-Ocaña [5], Ramón Alberto Batista-García [3,5,*] and Jorge Luis Folch-Mallol [1,*]

[1] Centro de Investigación en Biotecnología, Universidad Autónoma del Estado de Morelos, Cuernavaca 62209, Morelos, Mexico; amanda.martirena@uaem.edu.mx (A.M.-R.); jose.serrano@uaem.mx (J.G.S.-G.); mario.iza@uaem.edu.mx (M.L.I.-A.); maria.sanchez@uaem.mx (M.d.R.S.-C.)

[2] Centro de Ciencias Genómicas, Universidad Autónoma del Estado de Morelos, Cuernavaca 62209, Morelos, Mexico; yordaniz.perezlla@uaem.edu.mx

[3] Centro de Investigación en Dinámica Celular, Instituto de Investigación en Ciencias Básicas y Aplicadas, Universidad Autónoma del Estado de Morelos, Cuernavaca 62209, Morelos, Mexico; claribel.zenteno@uaem.edu.mx

[4] Facultad de Ciencias Químicas e Ingeniería, Universidad Autónoma del Estado de Morelos, Cuernavaca 62209, Morelos, Mexico

[5] Departamento de Biología Animal, Biología Vegetal y Ecología, Facultad de Ciencias Experimentales, Universidad de Jaén, 23071 Jaén, Spain; amocana@ujaen.es

* Correspondence: rabg@uaem.mx or rabg@ujaen.es (R.A.B.-G.); jordi@uaem.mx (J.L.F.-M.)

Table S1. Culture media used for the morphological characterization of the plant endophyte E_15.1.

**PDA**	**g/L**
Papa (infusion)	200
Dextrose	20
Agar	15
**MEA**	
Malt	20
Glucose	20
Peptone	1
Agar	15
**CYA**	
Saccharose	30
Yeast extract	5
K_2HPO_4	1
$NaNO_3$	0.3
KCl	0.05
$MgSO_4$	0.05
$FeSO_4$	0.001
$ZnSO_4$	0.001
$CuSO_4$	0.0005
Agar	15

Table S2. *A. brasiliensis* E_15.1 genome assembly statistics and general annotation features.

Total length (nt)	36’964,423
Total contigs	828
Average contig length (nt)	38,475.2
Longest contig (nt)	4’546,351
N50 sequence length (nt)	1’968,182
N90 sequence length (nt)	384,767
Predicted CDS’s	10,992
GC-content (%)	49.4

Table S3. Average Nucleotide Identity values for several A. brasiliensis reference strains including E-15.1 in the bottom line. Mb genome size in Megabases.

Genome	Genome	ANI %	Mb
*Asperguillus*_sp_E_15.1.fna	*Asperguillus_brasiliensis*_GCA_001889945.1.fna	96.8459	35.8
*Asperguillus*_sp_E_15.1.fna	*Asperguillus_brasiliensis*_GCA_027924045.1.fna	96.4616	36
*Asperguillus*_sp_E_15.1.fna	*Asperguillus_brasiliensis*_GCA_027924065.1.fna	96.876	36.1
*Asperguillus*_sp_E_15.1.fna	Asperguillus_brasiliensis_GCA_027924085.1.fna	96.9041	35.4
*Asperguillus*_sp_E_15.1.fna	*Asperguillus*_sp_E_15.1.fna	99	36.9
